# JMJD8 is a novel endoplasmic reticulum protein with a JmjC domain

**DOI:** 10.1038/s41598-017-15676-z

**Published:** 2017-11-13

**Authors:** Kok Siong Yeo, Ming Cheang Tan, Yat-Yuen Lim, Chee-Kwee Ea

**Affiliations:** 1 Institute of Biological Sciences, Faculty of Science, University of Malaya, 50603 Kuala Lumpur, Malaysia; 2 Department of Molecular Biology, University of Texas Southwestern Medical Center, Dallas, TX 75390-9148 United States

## Abstract

Jumonji C (JmjC) domain-containing proteins have been shown to regulate cellular processes by hydroxylating or demethylating histone and non-histone targets. JMJD8 belongs to the JmjC domain-only family that was recently shown to be involved in angiogenesis and TNF-induced NF-κB signaling. Here, we employed bioinformatic analysis and immunofluorescence microscopy to examine the physiological properties of JMJD8. We demonstrated that JMJD8 localizes to the lumen of endoplasmic reticulum and that JMJD8 forms dimers or oligomers *in vivo*. Furthermore, we identified potential JMJD8-interacting proteins that are known to regulate protein complex assembly and protein folding. Taken together, this work demonstrates that JMJD8 is the first JmjC domain-containing protein found in the lumen of the endoplasmic reticulum that may function in protein complex assembly and protein folding.

## Introduction

The Jumonji C (JmjC) domain-containing protein is a class of redox enzymes that catalyze protein hydroxylation or demethylation^1,2^. The JmjC domain was first classified by Takeuchi *et al*. in 1995^3^. Jumonji, which means cruciform in Japanese, was named after the observation of an abnormal cross-like neural groove that was formed in *jumonji* mutant embryos. Initially, it was thought that a JmjC domain usually co-exists with a JmjN domain in a non-adjacent manner in the same protein. However, a JmjC domain was later found to exist without a JmjN domain in various proteins that are conserved from bacteria to human^4,5^.

The JmjC domain-containing protein superfamily can be divided into two groups; histone demethylases that remove a methyl group from methylated lysine or arginine residues on the N-terminal tail of histones (KDM2-KDM7) and the JmjC domain-only proteins with ill-defined functions^1^. The majority of the histone demethylases contain additional protein domains that facilitate their interaction with histones. For example, Jmjd2C/GASC/KDM4C, a H3K9 specific demethylase^1,6^, contains a conserved TUDOR and a plant homeodomain (PHD) that bind methylated lysines or arginines. Interestingly, JMJD6 is the first member of the JmjC domain-only subgroup shown to function as a H3R4me2 (symmetrical)-specific histone demethylase^1,7^ and a histone lysine hydroxylase^8^. Importantly, recent studies have demonstrated that JmjC domain-containing proteins regulate numerous signaling pathways that are involved in cellular development, differentiation and proliferation. Perturbation of JmjC domain-containing protein expression is also associated with several human malignancies^3,9–14^.

JMJD8 is a member of the JmjC domain-only subgroup. Recent studies have shown that JMJD8 is involved in angiogenesis and cellular metabolism through interacting with Pyruvate kinase M2^15^. It is also a positive regulator of the TNF-induced NF-κB signaling pathway^16^. In this study, we examined the subcellular localization and the biophysical and biochemical properties of JMJD8. We found that JMJD8 contains a signal peptide and is mainly localized to ER lumen. The signal peptide of JMJD8 is important for its ER localization as well as its dimerization or oligomerization. Furthermore, we identified 35 potential JMJD8-interacting proteins that may shed light into understanding the biological function of JMJD8.

## Results

### JMJD8 contains a signal peptide that is essential for its ER localization

**Figure 1:**
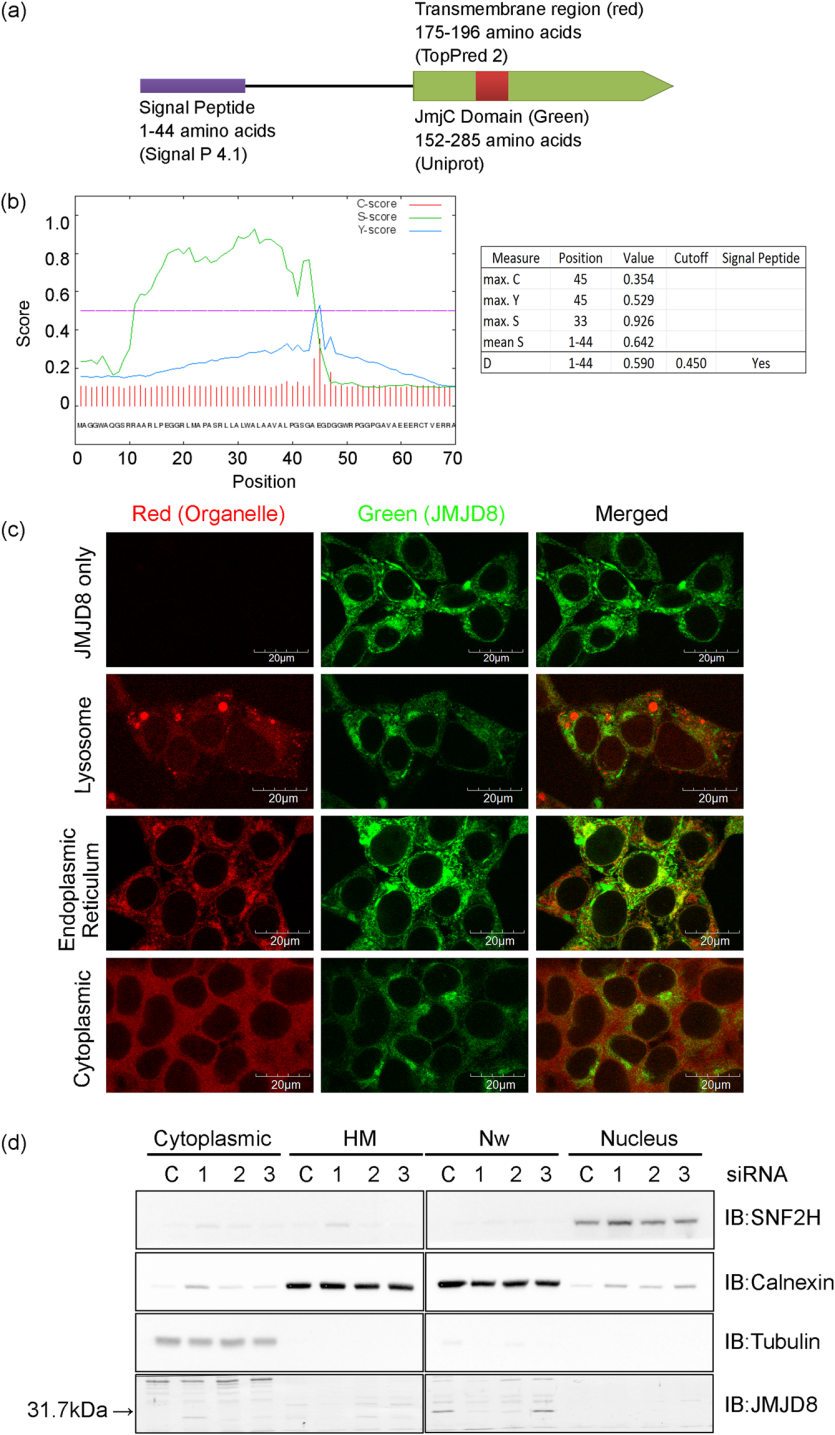
JMJD8 is an ER protein. (**a**) Schematic structure of JMJD8 predicted using indicated bioinformatics tools. (**b**) The protein sequence of JMJD8 obtained from NCBI was subjected to SignalP 4.1 analysis and three different scores were measured. The raw cleavage site score (C-score) is the output of the cleavage site prediction network, which is trained to distinguish signal peptide cleavage site; signal peptide score (S-score) is the output of signal peptide prediction network, which is trained to locate the signal peptides of a protein; and Y-score is a combination of C-score and the slope of S-score. In addition, mean S represented the average S-score of the possible signal peptide, whereas discrimination score (D-score) represented the mean S and maximum Y scores, which is used to discriminate signal peptide from the non-signal peptide. (**c**) HEK293T-JMJD8-eCFP stable cells were stained with LysoTracker (Lysosome), ER-tracker (Endoplasmic reticulum) or an anti-p65 antibody (Cytoplasmic). The yellow staining in the overlay image indicates colocalization of JMJD8 with ER. Images were acquired with an Olympus FV1000 confocal microscope. Scale bar: 20 μm. (n = 3). (**d**) HEK293T cells were transfected with either 10 nM control or an siRNA targeting JMJD8. Three siRNA oligos were tested to facilitate the identification of endogenous JMJD8 by immunoblotting. Cell lysates were prepared and fractionated into cytoplasmic, heavy membrane (HM-rich in lysosomes, ER, and mitochondria), nuclear wash (Nw-rich in ER) and nuclear fractions. The organelle specific proteins and JMJD8 were analyzed by immunoblotting with the indicated antibodies (Nuclear with SNF2h, ER with calnexin and cytoplasmic with tubulin). (n = 3). Full-length blots are presented in Supplementary Fig. S5.

To better understand the biochemical properties of JMJD8, we retrieved its protein sequence from NCBI (NM_001005920.2) and performed a sequence analysis (Fig. 1a). Using TopPred II analysis software^17^, we identified a putative transmembrane domain located between amino acid residues 175–196 of JMJD8. According to SignalP 4.1^18^, we obtained a discrimination score (D-score) of 0.590 at the position of 1–44 amino acid residues, which was above the default cut-off point of 0.450, suggesting that there is a signal peptide at the N-terminus of JMJD8 (Fig. 1b).

JmjC domain-containing proteins that function as histone demethylases are mainly localized to nucleus^1^. In contrast, the presence of a putative transmembrane domain and a signal peptide imply that JMJD8 may be a membrane bound protein localized to the cell membrane or other organelles. To determine the subcellular localization of JMJD8, we employed confocal microscopy to image HEK293T cells stably expressing JMJD8 fused with eCFP at the C-terminus (Fig. 1c). JMJD8 showed a distinct cytoplasmic staining that partially overlapped with the staining pattern of ER-Tracker™ Red dye, a fluorescent marker that specifically stains ER. JMJD8 did not colocalize with p65, a cytoplasmic protein, as well as other organelles, including lysosomes, nuclei, endosomes, Golgi bodies and mitochondria (Fig. 1c and see Supplementary Fig. S1). Interestingly, JMJD8 lacking the signal peptide (Δ45-JMJD8-eCFP) lost its ER localization and was distributed both in nucleus and cytoplasm, whereas the transmembrane domain deleted JMJD8 (ΔTM-JMJD8-eCFP) still localized to ER (see Supplementary Fig. S2).

To determine the subcellular localization of endogenous JMJD8, we carried out a crude subcellular fractionation experiment. First, we transfected HEK293T cells with three different siRNA oligos that target JMJD8 to facilitate the identification of JMJD8 by immunoblotting. Then, we lysed the cells in a hypotonic buffer and isolated the nuclear, cytoplasmic and heavy membrane (HM) fractions. HM fraction is enriched with lysosomes, ER and mitochondria^19^. However, we found that calnexin, an ER protein, was present in both HM and nuclear fractions. Some ER, especially rough ER, is connected to the outer nuclear membrane and thus may co-purified with nuclei^20,21^. To extract ER proteins from the nuclear fraction, we washed the nuclear pellets with a nuclear wash buffer. With this additional step, we were able to extract the majority of calnexin from the nuclear fraction without breaking the nucleus as SNF2H, a nuclear protein, was not extracted (Fig. 1d). Intriguingly, endogenous JMJD8 co-purified with calnexin in the nuclear wash fraction (Nw) but not in the HM fraction. In line with the endogenous JMJD8, ΔTM-JMJD8-eCFP is also enriched in the Nw fraction (see Supplementary Fig. S3).

To further determine if JMJD8 exists as an ER membrane or luminal protein, we employed a limited permeabilization and protease protection assay to determine the orientation of JMJD8 in the ER. ER integrity and protease efficiency were monitored by immunoblotting with ER membrane and lumen proteins. Interestingly, we noticed that similar to the luminal ER protein (PDIA3), JMJD8-FLAG-HA is resistant to proteinase K treatment in the protease protection assay compared to ER membrane proteins (Kinectin and MTDH), suggesting that it is an ER luminal protein (see Supplementary Fig. S4). In addition, we also identified three potential N-glycosylation sites located at asparagine residues 151, 161 and 230 using GlycoMine^22^ (see Supplementary Table S1) and shown that JMJD8 is sensitive to endoglycosidase H (endoH) digestion which suggested that JMJD8 is N-glycosylated (see Supplementary Fig. S4). Together, these results demonstrate that JMJD8 is a luminal ER protein and the signal peptide is required for its ER localization.

### Comparison between JMJD8 and other JmjC domain-containing proteins

**Figure 2:**
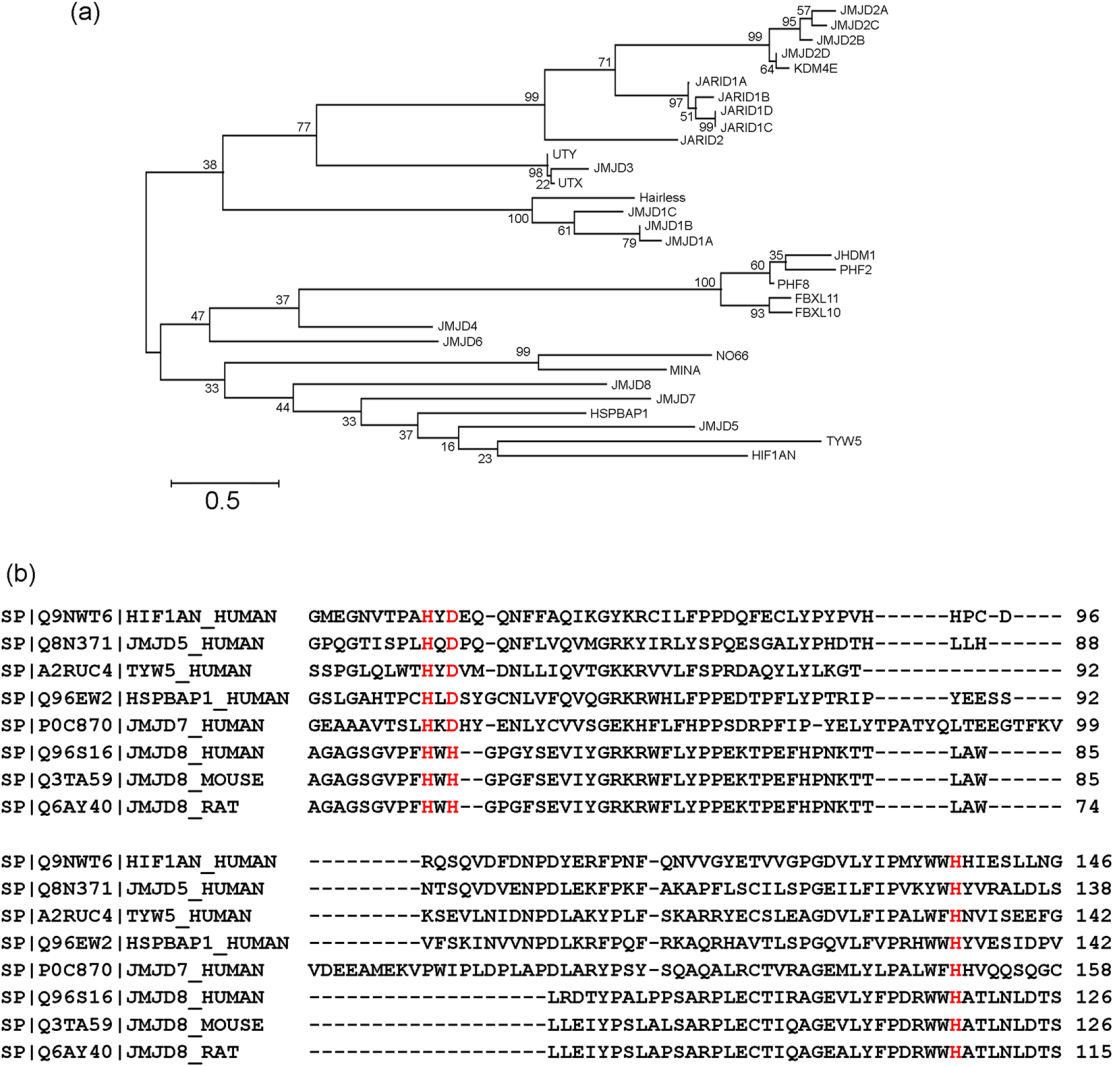
Phylogenetic analysis of JmjC domain-containing proteins. (**a**) Thirty-two sequences of JmjC domain-containing protein were retrieved from UniProtKB and a phylogenetic tree was generated using the Maximum Likelihood method. (**b**) Multiple sequence alignment of JmjC domains of HIF1AN, JMJD5, TYW5, HSPBAP1, JMJD7 and JMJD8 (human, mouse and rat). The Fe (II) binding site is highlighted in red.

Like JMJD6, JMJD8 contains only a JmjC domain without any other functional domain. To determine if JMJD8 clusters specifically with a particular group of JmjC domain-containing proteins, we employed a phylogenetic analysis to compare JMJD8 with 31 JmjC domain-containing proteins retrieved from UniProtKB. Interestingly, JMJD8 segregates into a cluster containing JMJD7, HSPBAP1, JMJD5, TYW5 and HIF1AN (Fig. 2a). Moreover, sequence alignment of JmjC domains showed that JMJD8 has a histidine, which is conserved among JMJD8 from different species, instead of an aspartic acid at its Fe (II) binding site (Fig. 2b). These results suggest that JMJD8 is distinct from typical JmjC domain-containing histone demethylases.

### Signal peptide of JMJD8 is essential for dimerization or oligomerization

**Figure 3:**
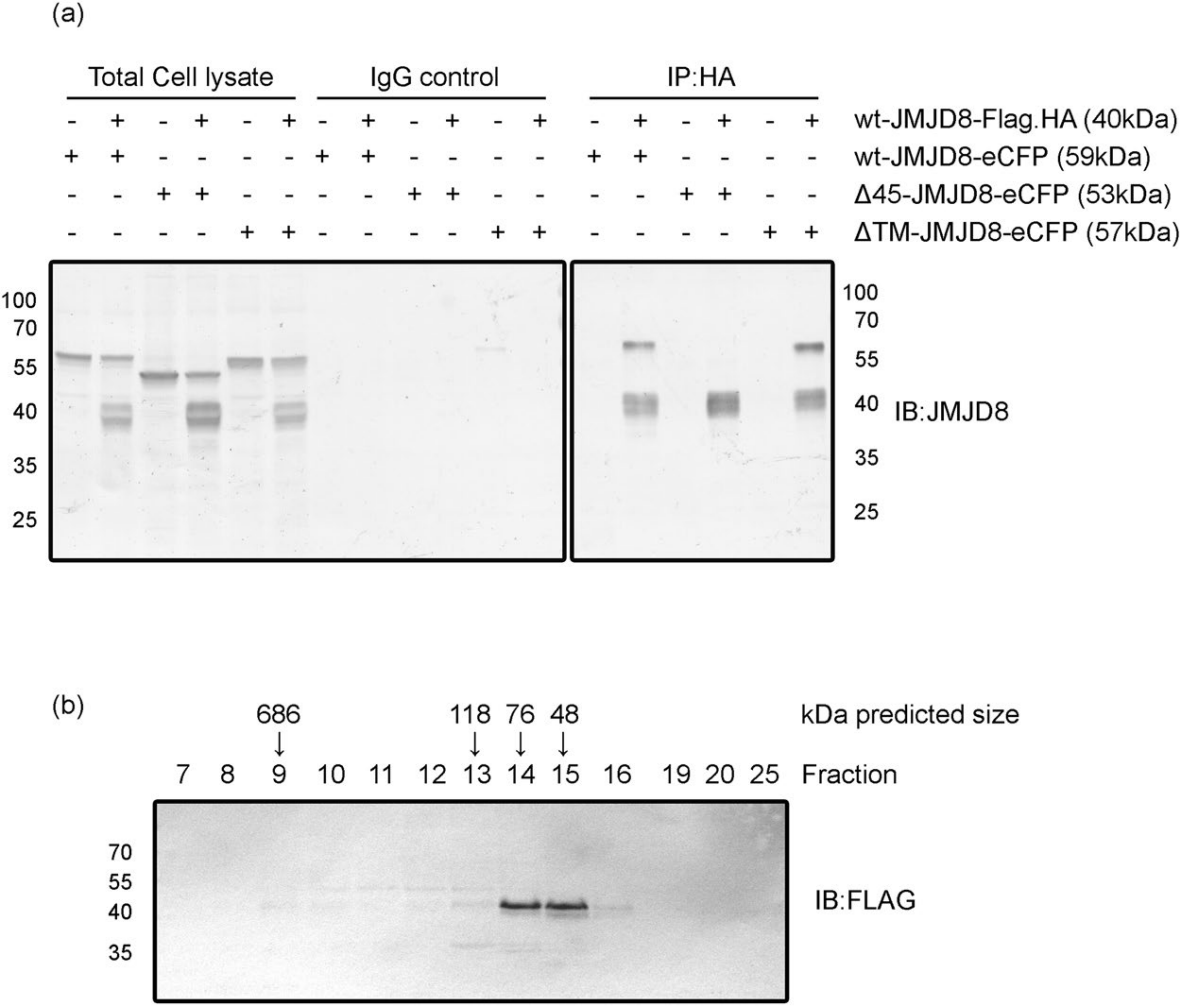
JMJD8 forms dimers or oligomers. (**a**) HEK293T cells were transfected with plasmids that express the indicated proteins. The interaction between wild-type and mutant JMJD8 was determined with an immunoprecipitation assay using an anti-HA antibody or mouse IgG. Total cell lysates and immunoprecipitated products were immunoblotted with the indicated antibodies (n = 3). (**b**) Total cell lysate from HeLa S3 cells stably expressing JMJD8-FLAG-HA (HeLa-JMJD8-FLAG-HA) was subjected to gel filtration (Superdex 200) analysis. The fractions were collected and immunoblotted with an anti-FLAG antibody. Full-length blots are presented in Supplementary Fig. S6.

To examine whether JMJD8 forms oligomer, we performed a co-immunoprecipitation assay. We co-expressed FLAG-HA-tagged and eCFP-tagged JMJD8 in HEK293T cells and immunoprecipitated JMJD8 with a HA-specific antibody followed by immunoblotting using a JMJD8-specific antibody. FLAG-HA-tagged JMJD8 interacted with eCFP-tagged wild type and ΔTM-JMJD8, but not with eCFP-tagged Δ45-JMJD8 (Fig. 3a). To further confirm the dimerization or oligomerization of JMJD8 *in vivo*, we performed a gel-filtration chromatography (Superdex 200) analysis of cell lysate prepared from HeLa S3 cells stably expressing a JMJD8-FLAG-HA (HeLa-JMJD8-FLAG-HA). Consistently, the majority of JMJD8 eluted from the gel filtration column in fractions presented as monomers and dimers in the cells, which corresponded to a molecular weight of 48 and 76 kDa, respectively (Fig. 3b). Moreover, a small population of JMJD8 eluted as high molecular weight species between 118–686 kDa (the estimated molecular weight for fraction 9–13). These results suggest that the signal peptide of JMJD8 is required for its dimerization or oligomerization.

### JMJD8 may be involved in protein complex assembly and protein folding

**Figure 4:**
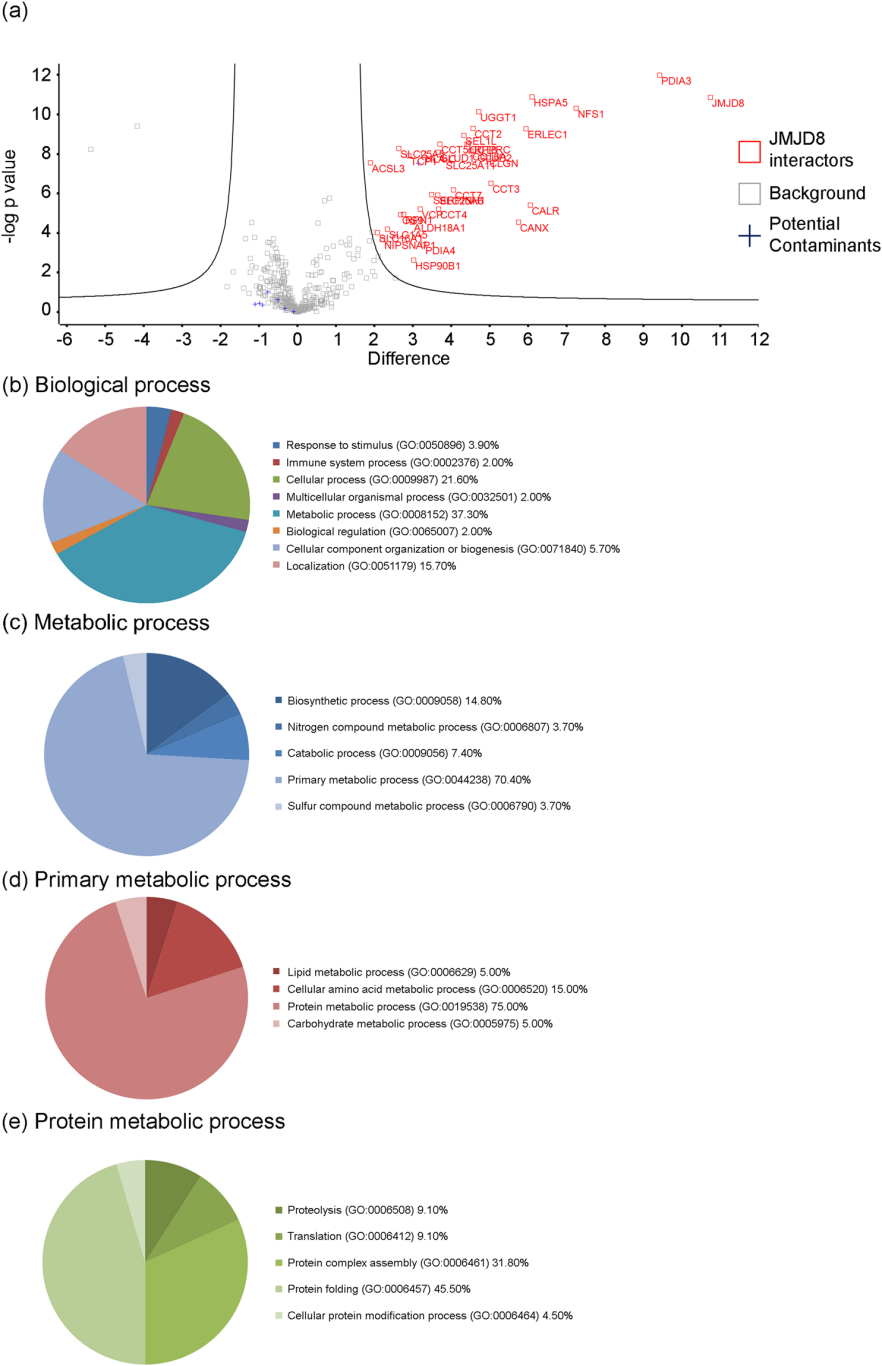
Interaction partners of JMJD8. Nuclear wash (Nw) fractions generated from HEK293T-JMJD8-FLAG-HA cells were subjected to immunoprecipitation using FLAG-agarose beads. The beads were digested and subjected to mass spectrometry analysis. (n = 6) (**a**) Volcano plot represented all identified targets. Statistically significant targets with a FDR < 0.01 and a fold change greater than 1.5 fold are shown in red. (**b**) Potential JMJD8 targets were subjected to gene ontology analysis. The pie chart was generated from the percent of gene hit against a total number of genes in process hits. Distribution of identified proteins was further divided into a subset of biological processes such as (**c**) metabolic processes, (**d**) primary metabolic processes and (**e**) protein metabolic processes.

In our previous study, we showed that JMJD8 functions as a positive regulator of the TNF-induced NF-κB pathway^16^. To better understand the biological functions of JMJD8, we sought to identify the interacting partners of JMJD8. We carried out an unbiased mass spectrometry analysis of proteins bound to JMJD8. Our subcellular fractionation assay showed that endogenous JMJD8 is enriched in the Nw fraction (Fig. 1d). Therefore, we subcellular fractionated HEK293T-JMJD8-FLAG-HA cells to obtain Nw fraction for immunoprecipitation to minimize undesired background protein and to target only ER-localized JMJD8. A number of JMJD8 potential interacting partners were identified (see Supplementary Table S2). By applying an established stringent filtering protocol as previously described by Kriegsheim’s group^23^, we identified 35 statistically significant targets that interact with JMJD8 in cells (Fig. 4a and see Supplementary Table S3). To determine if any biological processes are enriched among the 35 identified targets, we further examined all the 35 targets with PANTHER, a gene ontology analysis software^24^. Interestingly, the majority of the JMJD8 interaction partners were grouped under metabolic and cellular processes which are in line with a previous study^15^ (Fig. 4b and see Supplementary Table S4). According to PANTHER, we found that among the 35 proteins, 19 proteins are involved in metabolic processes (GO: 0008152) that can be further assigned to more detailed processes, including lipid metabolic process (GO: 0006629) (5%), cellular amino acid metabolic process (GO: 0006520) (15%), protein metabolic process (GO: 0019538) (75%) and carbohydrate metabolic process (GO: 0005975) (5%). Fifteen are classified into protein metabolic processes such as proteolysis (GO: 0006508) (9.1%), translation (GO: 0006412) (9.1%), protein complex assembly (GO: 0006461) (31.8%), protein folding (GO: 0006457) (45.5%) and cellular protein modification process (GO: 0006464) (4.5%). The majority of the target proteins clustered into protein complex assembly (7 targets) and protein folding (10 targets) (Fig. 4b–e and see Supplementary Table S4). These results demonstrate that JMJD8 may form complexes that regulate protein complex assembly and protein folding.

## Discussion

To the best of our knowledge, this is the first report to demonstrate that JMJD8 is a luminal ER localized JmjC domain-only protein (Fig. 1c and see Supplementary Fig. S4). We showed that JMJD8 contains an N-terminal signal peptide that is required for its ER localization (Fig. 1). Intriguingly, the signal peptide of JMJD8 is still intact in the mature protein based on the observed protein size after endoH treatment that matches the predicted molecular weight of JMJD8-FLAG-HA (39.9 kDa) (see Supplementary Fig. S4). There are examples of uncleaved signal peptide found on other proteins, such as CD18 and prion protein^25,26^. The uncleaved signal peptide of the prion protein tethers the protein to the ER membrane within the lumen. Thus, it is possible that the uncleaved signal peptide of JMJD8 anchors JMJD8 to the ER membrane with the rest of JMJD8 residing in the ER lumen. Consistent with this observation, a recent study by Boeckel *et al*. revealed that JMJD8 localizes to the extranuclear region instead of the nucleus. Interestingly, we previously demonstrated that JMJD8 is a positive regulator of the TNF signaling pathway^16^. In addition, ubiquitinated NF-κB signaling components, including RIP1, Bcl10 and TRAF2, have been shown to be recruited to the ER, which is required for optimal activation of NF-κB^19^. Therefore, our finding that JMJD8 is an ER protein further underscores the importance of ER as a signaling platform for the NF-κB pathway. However, further experimentation is required to test whether the ER localization of JMJD8 is important for its function. Sequence analysis of JMJD8 also predicted that JMJD8 contains a TM domain. However, deletion of TM domain does not affect the ER localization of JMJD8. It is possible that the prediction was inaccurate and JMJD8 is an ER lumen protein instead of ER membrane bound protein. Alternatively, the ΔTM-JMJD8 may form dimer or oligomer with endogenous JMJD8 and thus retain its ER localization.

Phylogenetic analysis suggests that JMJD8 belongs to a cluster containing JMJD7, HSPBAP1, JMJD5, TYW5 and HIF1AN (Fig. 2), which implies that JMJD8 may function similarly to this group of proteins. However, this group of JmjC domain-containing proteins has very diverse biological functions. For instance, TYM5 is a hydroxylase that is responsible for the biosynthesis of tRNA^phe^ ^27^ while HIF1AN is an asparaginyl hydroxylase that regulates the transcriptional activity of hypoxia-inducible factor^28^ and modifies ankyrin repeats in IκB proteins^29^. On the other hand, JMJD5 is a H3K36me2 histone demethylase that regulates p53 and cell proliferation^30–32^ whereas the biological function of JMJD7 and HSAPBAP1 remain to be identified.

A typical JmjC domain contains three conserved residues (H, E/D, H) that interact with Fe (II) and α-ketoglutarate^33^. Binding of these cofactors is important for the hydroxylase or demethylase activities of JmjC domain-containing proteins. In this regard, we suspect that JMJD8 may not be a hydroxylase or demethylase since the conserved aspartic acid or glutamic acid residue is substituted with a histidine residue in JMJD8, which may affect its binding to cofactors. However, it remains to be determined whether JMJD8 possesses any demethylase enzymatic activity which will be an exciting subject for future study.

Our co-immunoprecipitation and gel filtration chromatography studies suggest that JMJD8 forms predominantly monomers and dimers, and a small population of JMJD8 forms oligomers or high molecular weight complexes with other proteins (Fig. 3). Consistently, JMJD6 has been shown to form homo-oligomer in cells^34,35^. Moreover, the ER localization is required for dimerization or oligomerization of JMJD8 as Δ45-JMJD8, which localizes to cytoplasmic and nuclear compartments, fails to form dimers or oligomers.

Through the application of high throughput mass spectrometry analysis of JMJD8 bound proteins, we identified 35 statistically significant targets that are involved in various biological processes (Fig. 4). In line with the previous study by Boeckel *et al*., our data also suggest that JMJD8 interacting proteins are mainly involved in metabolic processes (Fig. 4b)^15^. In this regard, JMJD8 plays a role in angiogenesis as angiogenesis involves metabolism^15,36,37^. Furthermore, metabolism closely relates to the proliferation of cancer cells and is suppressed by JMJD8 knockdown^38,39^. Interestingly, ten target proteins (see Supplementary Table S4) belong to the category of protein folding suggesting that JMJD8 may form complexes with these chaperonins (T-complex protein 1)^40^ and calcium-binding proteins (Calnexin, Calmegin and Calreticulin)^41^ to modulate protein folding. However, we should be cautious when dealing with overexpression of an ER protein since the ER is a protein folding compartment and is sensitive to changes in protein homeostasis^42^. Ectopic expression of JMJD8 may overload the ER and lead to misfolding of JMJD8. Misfolded JMJD8 may interact with the cellular protein folding machinery, which may give rise to false positive interacting proteins that are irrelevant to the physiological function of JMJD8.

Consistent with the previous study by Boeckel and co-authors, we detected HSPA5, calnexin, and SEL1L as JMJD8 interacting partners. However, we are unable to detect pyruvate kinase M2 (PKM2) in our mass spectrometry analysis^15^. The difference in the identified targets may be attributed to the differences in the expression systems and cell lysates used in JMJD8 purification. In their study, JMJD8 was transiently expressed in HEK293 cells and total cell lysates were used for immunoprecipitation of JMJD8. On the contrary, our analysis focused on ER-bound JMJD8 because our biochemical fractionation of HEK293T cell extracts showed that majority of endogenous JMJD8 is localized to the ER that co-purified with the nuclear fraction (Fig. 1d). Among the 35 JMJD8 interacting proteins, 15 are ER proteins (lumen or membrane, see Supplementary Table S5), which are consistent with our findings that JMJD8 is a luminal ER protein.

In conclusion, our results reveal that JMJD8 is a JmjC domain-only protein that is localized to the lumen of endoplasmic reticulum. Moreover, the signal peptide is important for its ER localization and dimerization/oligomerization. In addition, JMJD8 may form protein complexes that are involved in protein folding.

## Methods

### Cell culture

HEK293T and HeLa S3 cells were maintained in Dulbecco’s modified Eagle’s medium (DMEM; Gibco) supplemented with 10% (v/v) FBS, 100 IU/ml penicillin and 100 µg/ml streptomycin (Gibco).

### Reagents and antibodies

Antibodies against p65 (C-20), SNF2H (H300), Tubulin (TU-02) and HA-probe (HA.C5) were acquired from Santa Cruz Biotechnology. Anti-JMJD8 and PDIA3 antibodies were purchased from Abnova. The anti-calnexin antibody was purchased from Abcam. Antibodies against EEA1 (C45B10), RCAS1 (D2B6N), AIF (D39D2), Kinectin 1 (D5F7J) and Lyric/Metadherin (MTDH) (2F11C3) were purchased from Cell Signaling Technology. LysoTracker® Red DND-99 and ER-Tracker™ Red dye were purchased from Invitrogen.

### Plasmid and expression vectors

The human JMJD8 transcript was amplified by polymerase chain reaction (PCR) from human cDNA and cloned into a pcDNA3 vector (Invitrogen) to generate pcDNA3-hJMJD8 with 2 × FLAG and 2 × HA or eCFP at the C-terminus. To generate Δ45-JMJD8 and ΔTM-JMJD8 mutants, full-length JMJD8 was used as a template for PCR with specific deletion primers for each construct. All constructs were verified by Sanger sequencing.

### JMJD8 localization assay

To examine the localization of JMJD8, HEK293T cells stably expressing JMJD8-eCFP were fixed with 4% formaldehyde for 15 minutes and then permeabilized and blocked with 1 × PBS supplemented with 5% fetal bovine serum and 0.3% Triton X-100 for 30 minutes. The cells were then incubated overnight with primary antibodies according to the manufacturer’s recommended dilution. Next, cells were washed three times with 1 × PBS followed by a 1-hour incubation with specific AlexaFluor-conjugated secondary antibodies (Cell Signaling). For detection of lysosome and endoplasmic reticulum, the cells were treated with either 25 nM LysoTracker® or 0.25 μM ER-Tracker™ for 15 minutes. Images were acquired with an Olympus FV1000 confocal microscope using a 100× objective lens. Images were analyzed using the cellSens standard and FV10-ASW viewer software (Olympus).

### Protease Protection assay

To examine the orientation of JMJD8, overnight culture of HEK293T cells stably expressing C-terminal FLAG-HA-tagged JMJD8 were washed two times with KHM buffer (110 mM Potassium acetate, 7.5 mM Magnesium Chloride, and 20 mM HEPES pH7.2) and permeabilized with 50 μg/ml of Digitonin for 5 minutes. Next, cells were washed three times with ice-cold KHM buffer and scraped in ice-cold KHM buffer. The cells were pelleted, split into three tubes and treated with or without 10 μg/ml of proteinase K at 37 °C for 5 minutes. NP40 was added to one of the tubes to solubilize organelle proteins followed by proteinase K treatement  to act as a positive control for protein digestion. Protease activities were terminated by the addition of 5 mM phenylmethylsulfonyl fluoride (PMSF). The samples were treated with SDS-sample buffer, boiled for 10 minutes, and subjected to immunoblotting analysis.

### Endoglycosidase H (EndoH) digestion assay

To verify whether JMJD8 undergoes N-glycosylation, immunoprecipitated JMJD8-FLAG-HA was denatured with glycoprotein denaturing buffer (New England Biolabs) at 95 °C for 10 minutes. Denatured protein was digested with or without EndoH (500U) at 37 °C for 1 hour. Then, the samples were treated with SDS-sample buffer, boiled for 10 minutes, and subjected to immunoblotting analysis.

### Bioinformatic analysis and phylogenetic tree generation

The protein sequence of JMJD8 was obtained from NCBI (NM_001005920.2) and subjected to signal peptide analysis (Signal P 4.1)^18^, transmembrane motif analysis (TopPred2)^17^ and GlycoMine^22^. The sequences of JmjC protein were retrieved from UniProtKB^43^ with the following accession numbers and amino acid regions: JHDM1A (Q9Y2K7; 148–316), JHDM1B (Q8NHM5; 178–346), JMJD1A (Q9Y4C1; 1058–1281), JMJD1B (Q7LBC6; 1498–1721), JMJD1C (Q15652; 2274–2498), JMJD2A (O75164; 142–308), JMJD2B (O94953; 146–309), JMJD2C (Q9H3R0; 144–310), JMJD2D (Q6B0I6; 146–312), JARID1A (P29375; 437–603), JARID1B (Q9UGL1; 453–619), JARID1C (P41229; 468–634), JARID1D (Q9BY66; 458–624), UTX (O15550; 1095–1258), JMJD3 (O15054; 1339–1502), PHF8 (Q9UPP1; 231–387), JHDM1D (Q6ZMT4; 230–386), TYW5 (A2RUC4; 102–267), HIF1AN (Q9NWT6; 142–312), PHF2 (O75151; 197–353), UTY (O14607; 1042–1205), JMJD4 (Q9H9V9; 188–347), JMJD5 (Q8N371; 271–416), JMJD6 (Q6NYC1; 141–305), JMJD7 (P0C870; 128–307), JMJD8 (Q96S16; 201–334), JARID2 (Q92833; 884–1048), KDM4E (B2RXH2; 143–309), HSPBAP1 (Q96EW2; 124–288), Hairless (O43593; 946–1157), MINA53 (Q8IUF8; 139–271), and NO66 (Q9H6W3; 294–439). The phylogenetic tree was generated according to the recommendation by Hall *et al*.^44^ using the Maximum Likelihood method based on the Le and Gascuel model^45^. The phylogenetic tree was drawn using MEGA6 with 32 amino acid sequences^46^.

### Immunoprecipitation

To examine the oligomerization of JMJD8, HEK293T cells were co-transfected with different JMJD8-expressing constructs. The cells were lysed in an IPKA lysis buffer (20 mM Tris pH 7.5, 150 mM NaCl, 10% glycerol, 25 mM β-glycerol-phosphate, 1 mM sodium orthovanadate, 1 mM DTT, 1 mM PMSF, and 1% Triton ×100) and the total protein concentration was quantitated using the Bradford assay. Five hundred micrograms of total protein was immunoprecipitated with anti-HA or mouse IgG antibodies with 10 µl of 50% slurry protein A/G beads (Pierce, USA) overnight at 4 °C. Beads were then washed four times with the IPKA lysis buffer. Efficiency of the pull-down assay was verified by immunoblotting using the indicated antibodies. Thirty micrograms of total cell lysate were included as a positive control.

### Sample preparation for mass spectrometry

To examine the interaction partner of JMJD8, the cells were first fractionated to obtain the nuclear wash (Nw) fraction. Briefly, HEK293T-JMJD8-FLAG-HA cells were washed three times with 1 × PBS and lysed with a hypotonic lysis buffer (10 mM Tris, pH 7.5; 1.5 mM MgCl_2_; 10 mM KCl; 0.5 mM DTT; 0.5 mM PMSF and 1× Protease Inhibitor). The nuclear pellet was isolated by centrifugation at 500 g, 4 °C for 5 minutes. The pellet was washed once with hypotonic lysis buffer, resuspended in a Nw buffer (50 mM Tris pH 7.5; 10 mM MgCl_2_; 250 mM Sucrose; 0.2% NP40; 0.5 mM DTT; 0.5 mM PMSF and 1× Protease Inhibitor), and rotated at 4 °C for 1 hour. The Nw fraction was obtained via centrifugation at 500 g, 4 °C for 5 minutes. Protein concentration of the Nw fraction was quantified using the Bradford assay. Three miligrams of Nw fraction was precleared with 60 μl of A/G beads (Pierce, USA) and subjected to immunoprecipitation as previously described with FLAG-agarose beads (Pierce, USA). The beads were washed three times with TBS containing 0.5% NP40 and two more times with TBS only. On-bead digestion protocol adapted from Turriziani, B. *et al*.^23^ was used to denature and digest the immunoprecipitated proteins. Briefly, 60 μl of buffer 1 (50 mM Tris pH 7.5; 2 M Urea; 5 ng/ul Trypsin) was added to the washed beads and incubated for 30 minutes at 27 °C with shaking at 800 rpm. The supernatant was collected via centrifugation at 1000 g for 1 minute. Next, the beads were washed twice with 25 μl buffer 2 (50 mM Tris, pH 7.5; 2 M Urea; 1 mM DTT) and the supernatant pooled together with buffer 1. The sample was then kept at room temperature for overnight digestion. After digestion, 20 μl of 5 mg/ml Iodoacetamide (IAA) was added to the mixture and incubated at room temperature in the dark for 30 minutes. The reaction was stopped by the addition of 1 μl of 100% trifluoroacetic acid (TFA). The reaction mix was desalted with a C18 spin column as per manufacturer’s recommendation. The eluates were speedvac-concentrated and resuspended with 35 μl of 0.1% formic acid and analyzed by mass spectrometry.

### Mass Spectrometry

All samples were analyzed with a Thermo Orbitrap Fusion Tribrid mass spectrometer (Thermo Scientific, San Jose, CA) equipped with a Thermo EASY-nanoLC system (Thermo Scientific, San Jose, CA) and a nanoelectrospray source. Five microliters of sample were injected into an Acclaim™ PepMap™ 100 C18 LC Column (Thermo Scientific, San Jose, CA) and separated with the Thermo EASY-nanoLC system loaded with a Thermo Scientific™ EASY-Spray™ C18 LC Column (Thermo Scientific, San Jose, CA) (2 μm, 75 μm × 50 cm). The samples were separated at a flow rate of 250 nl/min over 80 minutes with a gradient from 5% to 95% buffer B (99.9% Acetonitrile/0.1% formic acid). The raw data were collected continuously with a mass spectrometer in a data-dependent manner. A survey scan was recorded in the Orbitrap analyzer with a 60,000 resolution over a mass range between m/z 400–1600 Da and an automatic gain control (AGC) target at 4.0e^5^. Then, it was followed by the second stage of higher-energy collisional dissociation (HCD) MS/MS scans to the top 20 most intense ions from the survey scan with an AGC target of 1e^3^, a signal threshold of 1,000, auto scan range mode and 28% of HCD collision energy. Charge state was assigned to focus on ions that have a charge state of +2 and +3. Dynamic exclusion was enabled for 30 seconds repeat duration and 20 seconds exclusion duration with a repeat count of 3.

### Analysis of mass spectrometry data

The raw files generated from the mass spectrometer were analyzed with a MaxQuant software package^47^. The raw files were scanned against human-reviewed protein database (UniProtKB: Taxonomy number: 9606) with a Maximum error tolerance (ppm) of 20 ppm for the first search and a 6 ppm for the main search with a false discovery rate (FDR) of 0.01. Every raw file was considered as an independent experiment and the replicates were group together for subsequent statistical analysis. Carbamidomethylation for cysteine residue was set as fixed modification whereas methionine oxidation and acetylation of N-termini of proteins were defined as variable modification. The analyzed data were subjected to statistical analysis using Perseus^48^. A volcano plot was generated from student t-test with a FDR of 0.01 and a S0 of 1.5. The positive hits of JMJD8 interacting partners were further analyzed with the PANTHER classification system Version 11.1^24^.

## Electronic supplementary material

Supplementary materials

Supplementary table S2

Supplementary table S3

Supplementary table S4

Supplementary table S5
