# Supplementary material for: JMJD8 is a novel endoplasmic reticulum protein with a JmjC domain
Source: Sci Rep. 2017 Nov 13;7:15407. doi: 10.1038/s41598-017-15676-z (PMC5684140; doi:10.1038/s41598-017-15676-z)
Supplement: Supplementary file 1 — Supplementary materials [file 41598_2017_15676_MOESM1_ESM.pdf]

Supplementary Materials

JMJD8 is a novel endoplasmic reticulum protein with a JmjC domain.

Kok Siong Yeo [1], Ming Cheang Tan [1], Yat-Yuen Lim [1,*], Chee-Kwee Ea [1,2*]

Author information

Affiliations

1 Institute of Biological Sciences, Faculty of Science,

University of Malaya,

50603 Kuala Lumpur, Malaysia

2 Current address: Department of Molecular Biology,

University of Texas Southwestern Medical Center,

Dallas, TX 75390-9148, United States

*To whom correspondence should be addressed:

yatyuen.lim@um.edu.my; Chee-Kwee.Ea@utsouthwestern.edu

Phone: 603-79677022ext2587, Fax: 603-79557727

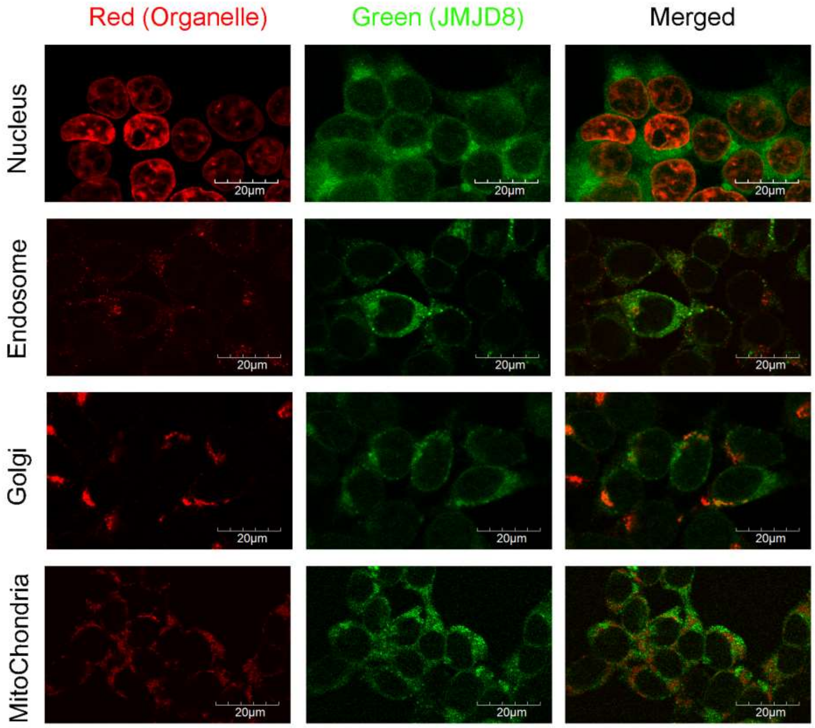

Supplementary Figure S1: Localization of JMJD8. Localization of JMJD8 in HEK293T-JMJD8-eCFP stable cells were visualized by immunofluorescence microscopy co-stained with organelle specific antibodies such as EEA1 (C45B10) (Cell signaling Tecnology, USA) for endosomes, RCAS1 (D2B6N) (Cell signaling Tecnology, USA) for Golgi, and AIF (D39D2) (Cell signaling Tecnology, USA) for mitochondria. Images were acquired with an Olympus FV1000 confocal microscope. Scale bar: 20 μm. (n = 2).

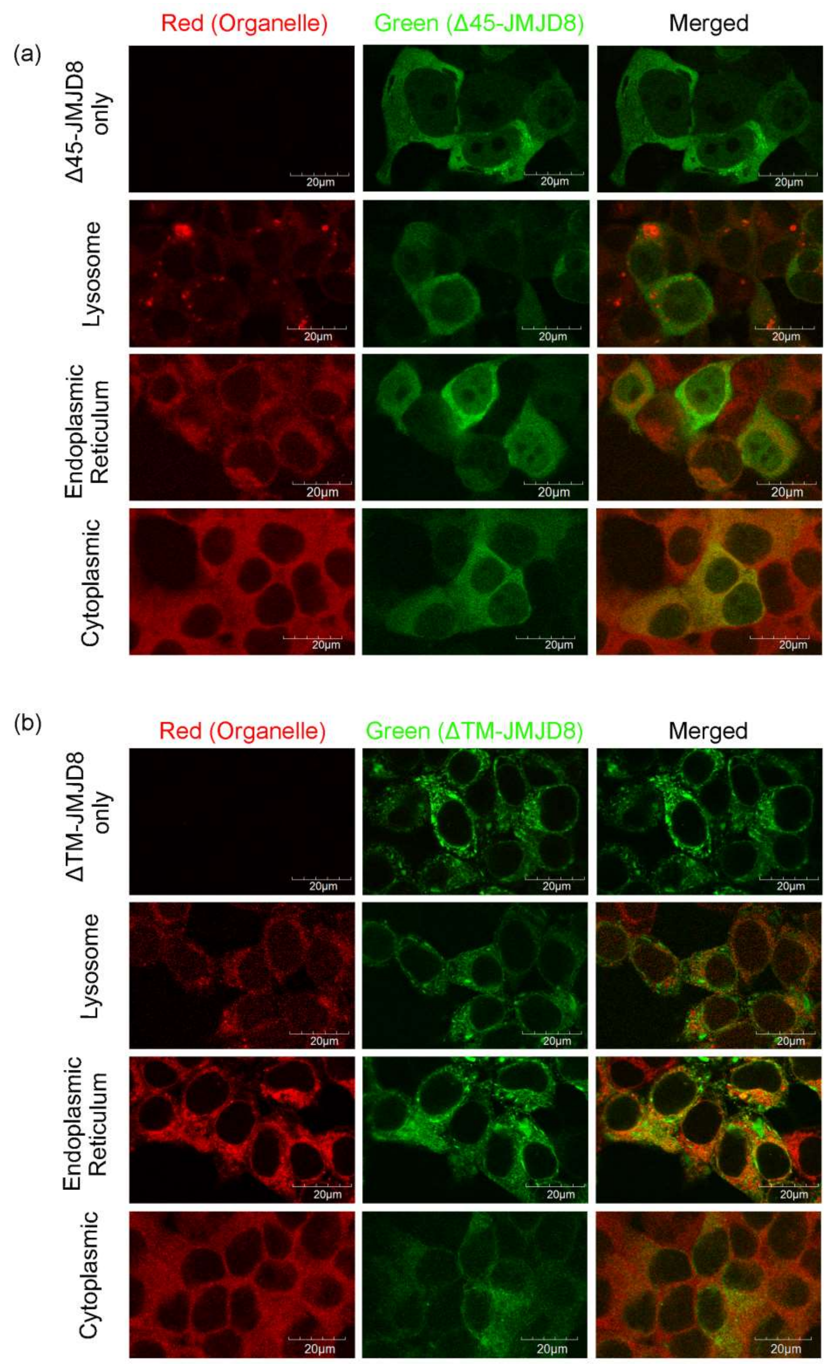

Supplementary Figure S2: Localization of JMJD8 mutants. (a) Localization of Δ45-JMJD8 and (b) ΔTM-JMJD8 in HEK293T stable cells were visualized and compared with LysoTracker-(Lysosome), ER-tracker-(Endoplasmic reticulum) and p65- (Cytoplasmic) immunofluorescence staining. Images were acquired with an Olympus FV1000 confocal microscope. Scale bar: 20 μm. (n = 3).

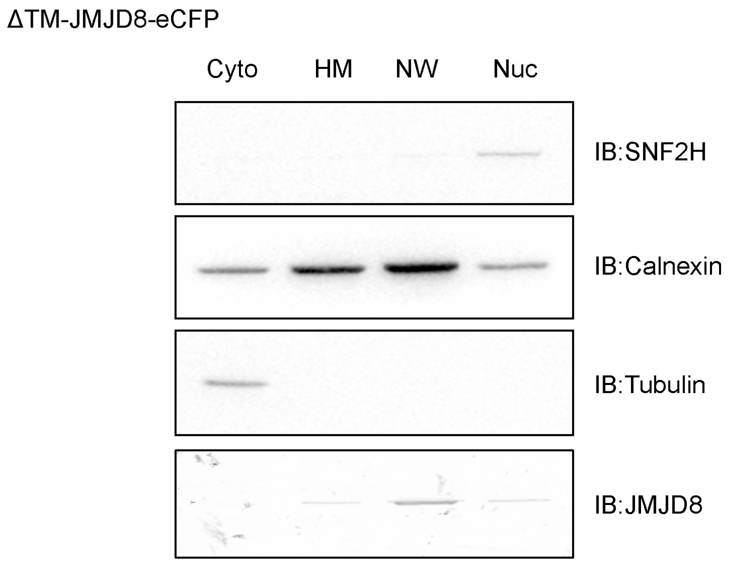

Supplementary Figure S3: Subcellular fractionation of ΔTM-JMJD8-eCFP mutants. Cell lysates from ΔTM-JMJD8-eCFP expressing HEK293T stable cells were prepared and fractionated into cytoplasmic, heavy membrane (HM-rich in lysosomes, ER, and mitochondria), nuclear wash (Nw-rich in ER) and nuclear fractions. The organelle specific proteins and JMJD8 were analyzed by immunoblotting with the indicated antibodies (Nuclear with SNF2h, ER with calnexin and cytoplasmic with tubulin). (n = 3).

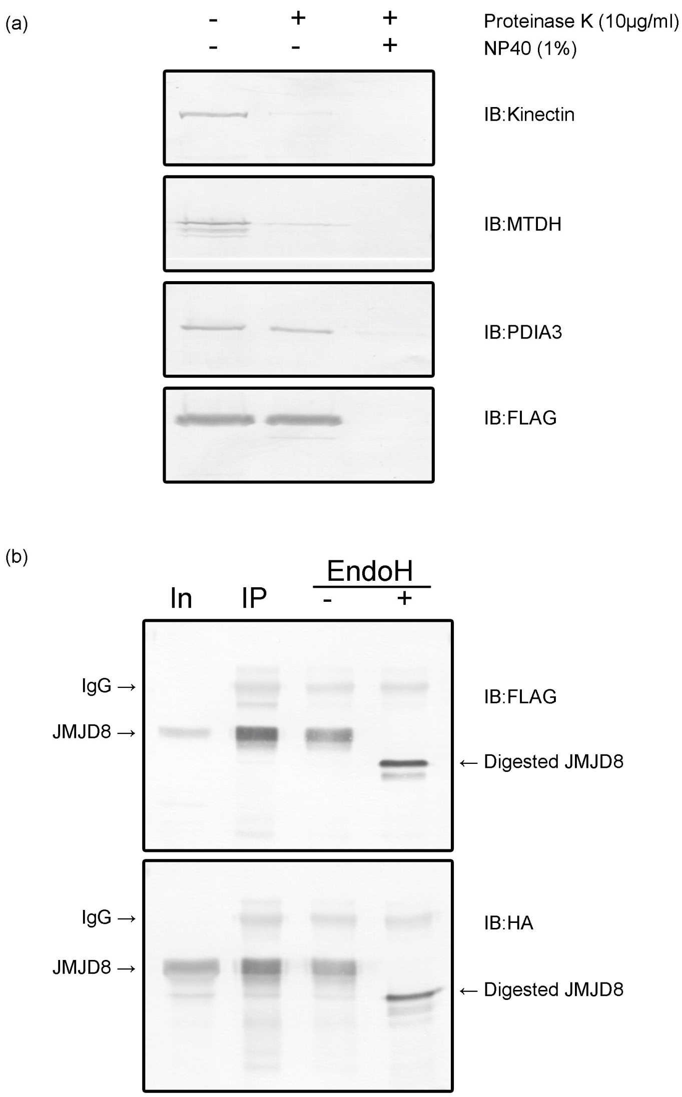

Supplementary Figure S4: Protease protection assay and EndoH sensitivity assay. (a) HEK293T cells stably expressing JMJD8-FLAG-HA were permeabilized with 50 μg/ml Digitonin for 5 minutes and treated with or without 10 μg/ml of proteinase K. Cells were solubilized with 1% of NP40 as a positive control for proteinase K digestion. ER membrane proteins and JMJD8 were analyzed by immunoblotting with the indicated antibodies (Kinectin and MTDH are ER membrane proteins, while PDIA3 is a luminal ER protein). (n = 2). (b) Immunoprecipitated JMJD8-FLAG-HA was subjected to EndoH digestion at 37°C for 1 hour. JMJD8 were analyzed by immunoblotting with the indicated antibodies. (n = 2).

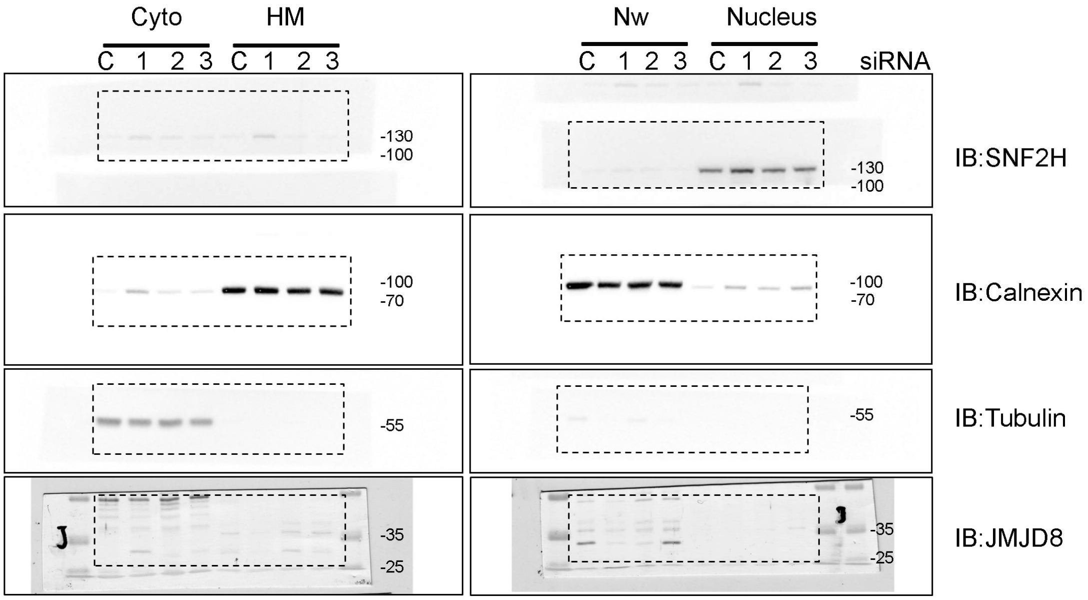

Supplementary Figure S5: Full-length images of immunoblots shown in Figure 1d.

(a)

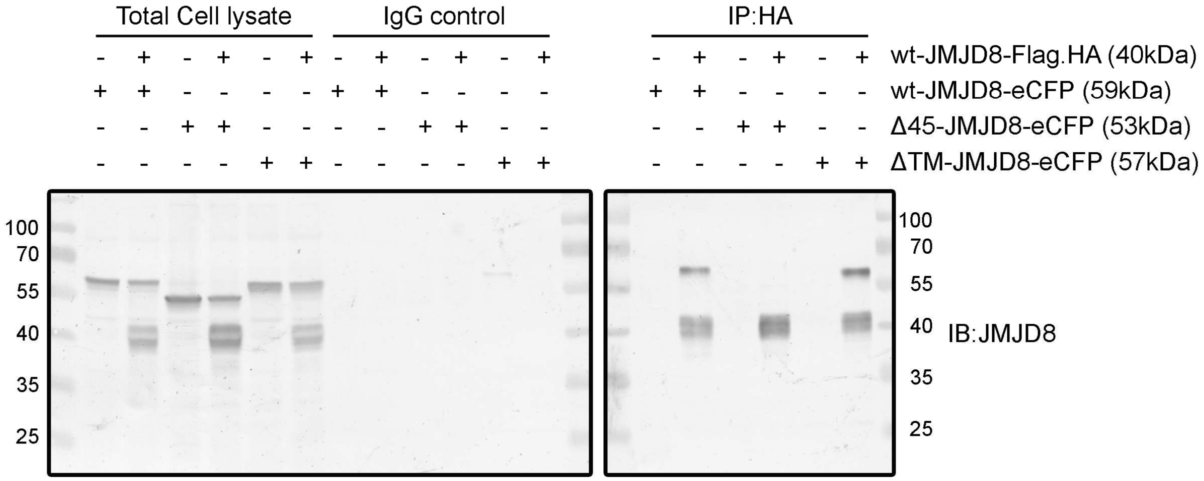

(b)

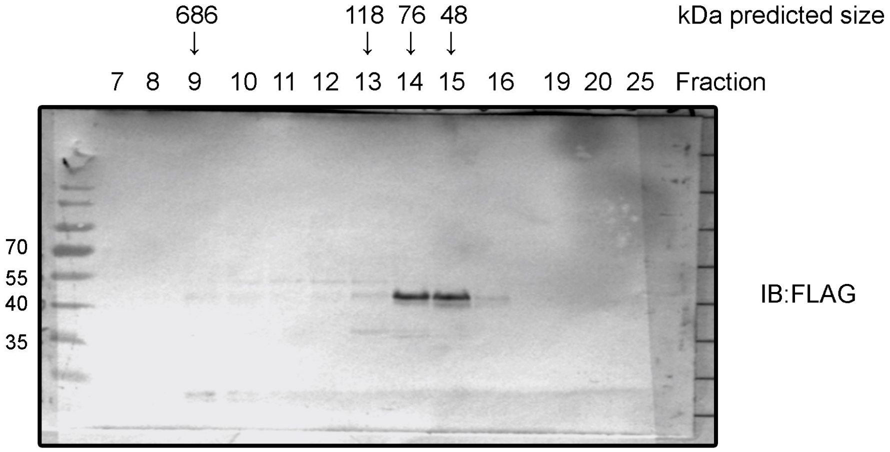

Supplementary Figure S6: Full-length images of immunoblots shown in Figure 3b.

Supplementary Table S1: N-glycosylation prediction with GlycoMine for JMJD8.

Protein	Position	Adjacent residues	Probability
JMJD8	151	QDPTSLGNDTLYFFG	0.823
JMJD8	161	LYFFGDNNFTEWASL	0.823
JMJD8	230	KTPEFHPNKTTLAWL	0.823
JMJD8	122	VVRLSTANTYSYHKV	0.086
JMJD8	160	TLYFFGDNNFTEWAS	0.074
JMJD8	273	RWWHATLNLDTSVFI	0.043
JMJD8	95	ILQGLTDNSRFRALC	0.018
